# Enhancing field GP engagement in hospital-based studies. Rationale, design, main results and participation in the diagest 3-GP motivation study

**DOI:** 10.1186/1471-2296-13-63

**Published:** 2012-06-21

**Authors:** Christophe Berkhout, Marie Vandaele-Bétancourt, Stéphane Robert, Solène Lespinasse, Gamil Mitha, Quentin Bradier, Anne Vambergue, Pierre Fontaine

**Affiliations:** 1 Department of General Medicine, University of Lille Nord de France – Lille 2, Faculty of Medicine, 59045, Lille Cedex, France; 2 Department of Endocrinology, Clinique Médicale, Claude Huriez Hospital, Regional and Academic Hospital Centre, 2 avenue Oscar Lambret, 59037, Lille Cedex, France

**Keywords:** General practice, Biomedical research, Behavioural research, Research design, Cooperative behaviour, Social identification

## Abstract

**Background:**

Diagest 3 was a study aimed at lowering the risk of developing type 2 diabetes within 3 years after childbirth. Women with gestational diabetes were enrolled in the study. After childbirth, the subjects showed little interest in the structured education programme and did not attend workshops. Their general practitioners (GPs) were approached to help motivate the subjects to participate in Diagest 3, but the GPs were reluctant. The present study aimed to understand field GPs’ attitudes towards hospital-based studies, and to develop strategies to enhance their involvement and reduce subject drop-out rates.

**Methods:**

We used a three-step process: step one used a phenomenological approach exploring the beliefs, attitudes, motivations and environmental factors contributing to the GPs’ level of interest in the study. Data were collected in face-to-face interviews and coded by hand and with hermeneutic software to develop distinct GP profiles. Step two was a cross-sectional survey by questionnaire to determine the distribution of the profiles in the GP study population and whether completion of an attached case report form (CRF) was associated with a particular GP profile. In step three, we assessed the impact of the motivation study on participation rates in the main study.

**Results:**

Fifteen interviews were conducted to achieve data saturation. Theorisation led to the definition of 4 distinct GP profiles. The response rate to the questionnaire was 73%, but dropped to 52% when a CRF was attached. The link between GP profiles and the rate of CRF completion remains to be verified. The GPs provided data on the CRF that was of comparable quality to those collected in the main trial. Our analysis showed that the motivation study increased overall participation in the main study by 23%, accounting for 16% (24/152) of all final visits for 536 patients who were initially enrolled in the Diagest 3 study.

**Conclusions:**

When a hospital-led study explores issues in primary care, its design must anticipate GP participation early in the trial. Based on our questionnaire response rates, we found that one in two GPs were willing to participate in our hospital-led study, regardless of their initial attitudes.

## Background

In France, general practice (GP) was recognized as an academic discipline in 2004, and the first GPs were graduated at the PhD level in 2010. While GP research networks and colleges already existed, GPs were not collaborating with academic research teams and they rarely published in peer-reviewed journals. On the academic side, working with GPs on clinical research was unfamiliar to hospital-based medical specialists. In particular, the hospital-based researchers were not used to approaching field GPs to participate in their research. We developed this study to understand the barriers to collaborative research by hospital-based specialists and field GPs in France. While the study’s conclusions might be obvious in countries where collaborative studies are regularly performed by both GPs and medical specialists (e.g., the United Kingdom, the United States, Canada, Australia, New Zealand, and the Netherlands), there are many countries like France where the work of GPs lies outside the university or where academic general practice is still developing. This study sheds light on the attitudes of field GPs towards academic medical specialists and academic research, with the goal of finding new ways to perform collaborative work. Our study was initiated after an academic hospital-led study, Diagest 3, experienced a significant dropout rate and attempts to engage field GPs to encourage their patients’ participation in the study failed.

Previously, the Diagest 2 observational cohort study [1] demonstrated that women with gestational diabetes (GD) have an increased risk of developing type 2 diabetes (T2D). The aim of the Diagest 3 study was to lower this risk by 30% through the implementation of a structured educational programme after childbirth. This open-label, uncontrolled, multicentre hospital-based cohort study was led by the Endocrinology Department of the Regional University Hospital of Lille, in Northern France. It enrolled pregnant women with GD and at least one additional risk factor (Registration references: PHRC 2003/R1907 and PROM 04-06-852, Ethical approval: CCPPRB Lille Jul. 6, 2004, Reference number 04/50). The number of subjects needed to fulfil the main outcome was 630, taking into account an expected dropout rate of 25%. Follow-up clinics were carried out in hospitals by endocrinologists at years 1, 2 and 3 after childbirth. At their first clinic, patients’ attitudes were often doubtful regarding the reality of their risk of developing T2D within 3 years. It was soon observed that 50% of the subjects, far greater than the 25% expected dropout rate, withdrew from the study, essentially deserting the hospital-led workshops.

One year after the study began, the Diagest 3 steering committee approached the GPs who had been identified by the study subjects as their “regular doctor” (compulsory registration with a GP practice was not yet the norm in France). These practitioners had already received an information sheet at the time their patient was enrolled in the study. Though not included in the original Diagest 3 study protocol, the GPs were sent an additional follow-up letter explaining the benefit of the educational workshops for their patients and they were invited to attend meetings with the study team. Only three of the invited GPs attended the meetings. In addition, some of the endocrinologists participating in the study independently sent letters to the GPs who usually referred patients to them, asking for biological data on the study patients. The response rate was below 10% (because it was off-protocol, precise data were not collected by the Diagest 3 investigators).

The Department of General Medicine was informed of the problems encountered in engaging GP support for the Diagest 3 study. We then initiated a motivation study, called Diagest 3-GP, to understand the obstacles to GP commitment to the hospital-based Diagest 3 study and reveal strategies to increase the number of patients finishing the study. The motivation study was added as a protocol amendment to the main Diagest 3 study. We identified 5 aims:

· To explore the beliefs and attitudes of Diagest 3 GPs that could explain their lack of involvement,

· To define profiles of attitudes towards the Diagest 3 study that differentiate GPs based on their motivation to engage,

· To study the distribution of these profiles in the Diagest 3-GP population,

· To determine the possibility of predicting the commitment of GPs based on these profiles, and

· To measure the impact of the motivation study on participation in the Diagest 3 study.

The Diagest 3-GP study was not intended to be interventional, as there is no comparator except the previous experience of the Diagest 3 team to involve GPs. Nevertheless, we raised the hypothesis that our observational and analytical work in the Diagest 3-GP study would have some impact on the subsequent participation of GPs in Diagest 3. Indeed, we expected that the Diagest 3-GP study would create a different situation of commitment where GPs would feel free to engage the study, compared to a situation where engagement could be considered as imposed by a superior authority (hospital medical specialists) [2].

## Methods

We found very little information in our review of the medical literature on the topic of field GP commitment to participate in hospital-led research, with most studies based on cross-sectional surveys using questionnaires. Many studies have investigated GP motivations in joining research networks as investigators [3], but none explored the beliefs, attitudes, motivations and environmental factors involved when GPs are asked to participate in research initiated by others and in which one of their patients has been enrolled. A search of the literature in the psychology of commitment revealed studies of how people make a commitment, or how a doctor can boost his or her patients’ compliance, but there were no papers addressing this issue specifically for GPs and hospital-led studies. Furthermore, GP social attitudes and behaviours have been described by many sociologists but not in this context.

Because of the lack of published literature, we developed a 3-step approach to assess the factors that affect field GP engagement in hospital-led studies. First, to explore the lack of GP interest that was experienced in the Diagest 3 study, we performed a qualitative analysis based on grounded theory to define distinct GP profiles; second, we conducted a cross-sectional survey based on these profiles to assess their distribution in our study population of Diagest 3-GPs; and finally, we performed process analysis to evaluate whether our work in the Diagest 3-GP motivation study had enhanced GP engagement in the main Diagest 3 study.

### Step one: Qualitative exploratory study

As the current literature did not provide clues as to the best way to explore GP beliefs and attitudes that might result in indifference or hostility to the Diagest 3 study, we found that it was necessary to build a working hypothesis on the most rigorous methodology. It was essential that our working hypothesis be scientifically innovative within the field of social sciences, supported by evidence based on an inductive approach, and deeply rooted in the research field. The most suitable method [4] appeared to be a grounded theory approach as initially described by Glaser and Strauss [5-7] and synthesized by Fernandez [8]. As our Diagest 3-GP research team included GPs, we wanted to exclude the possibility of any preconceptions and developed this step as rigorously as possible. Our approach involved a 4-stage procedure: sampling, data collection, coding and conception of one or more theories.

### Sampling

**Figure 1:**
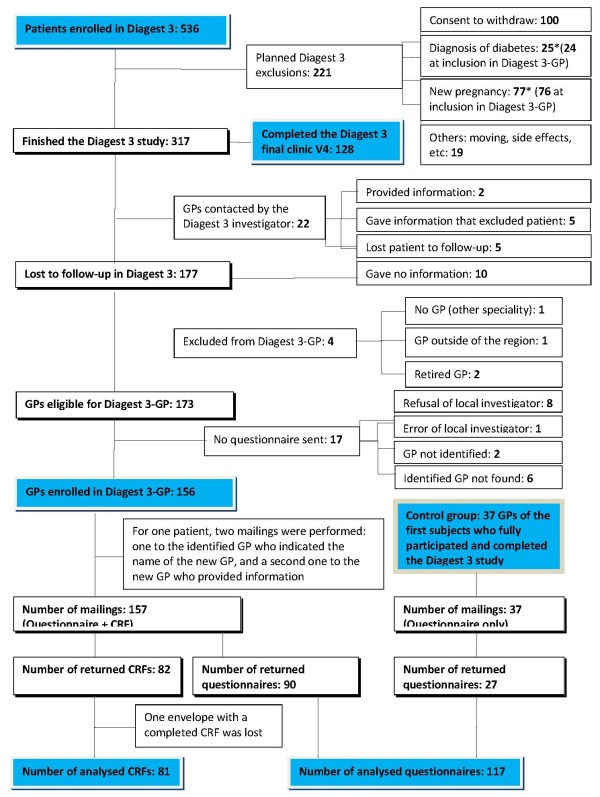
**Study participant distribution.** *One pregnancy and one diagnosis of diabetes were discovered by the local investigator of Diagest 3, though questionnaires for Diagest 3-GP had already been sent to these patients’ GPs. Neither of the GPs returned the questionnaires. The red arrow indicates 10 GPs who were contacted but did not reply; these GPs were reintegrated into the Diagest 3-GP study population. GP = general practitioner; V4 = final clinic visit (3 years after inclusion).

The study population consisted of GPs who had been identified as the regular doctor of the Diagest 3 patients who had withdrawn from the study (n = 177; Figure 1). To be eligible, GPs had to be running a practice in Northern France. The order of inclusion in the motivation study was the order of inclusion of their patients in the main study. The first 15 doctors received a letter from the Department of General Practice inviting them to participate in a face-to-face tape-recorded interview. Each doctor was called by one of the junior researchers who would be interviewing them (MVB, SL, SR), to verify that they met the inclusion criteria and to arrange a convenient time for the interview. In case of refusal or mismatch with the inclusion criteria (GP outside Northern France, retired, non-GP), the next doctor on the list was approached.

### Data collection

Semi-structured face-to-face interviews were conducted by 3 junior researchers (MVB, SR, SL) using a topic list. To avoid interviews being biased by the preconceived attitudes of the interviewing researchers, who were themselves GP trainees, the researchers knew as little as possible about the main study. One of the interviewers’ tasks was to gather as much information as they could about Diagest 3 from the interviewed GPs. Interviews were performed until theoretical data saturation plus at least two interviews for validation. Theoretical data saturation, as described by Glaser and Strauss, was reached when axial coding of the last transcript provided no new useful data to elaborate on the theory. Focus groups were also planned to cross-check data saturation and to explore a larger range of social attitudes. All interviews were digitally audiotaped and nonverbal expressions were noted by the interviewer. The recordings were transcribed by the interviewer with annotations of the significant nonverbal information. Transcripts were not submitted to the interviewees for acceptance.

### Coding

The transcripts were coded independently by 2 researchers who had not conducted the interviews (MVB, SR, SL). After open coding and axial coding, the research team met to compare outcomes. Discordance was resolved by consensus or by a third coder (CB). The first 9 transcripts were coded manually. Starting from the 10th interview, coding was performed using hermeneutic software (NVivo 8, QSR International Ltd., Southport, UK). Table 1 summarises the main characteristics of each interviewed GP, who conducted the interview, who coded the transcript, and whether a third coding was needed.

**Table 1:** Characteristics of interviewed GPs and the interview approach

**GP number**	**Age**	**Gender**	**Continuous education**	**Research**	**Interviewer**	**Coding**	**3rd coding**
1 (retired)		Male					
2	52	Male	Yes	No	SL	MVB, SR	
3 (refused interview)		Male					
4 (gynaecologist)		Female					
5	56	Male	No	No	SL	MVB, SR	
6 (refused to answer)		Male			SR		
7	47	Male			SR	MVB, SL	
8	46	Male	No	No	SR	MVB, SL	
9	39	Female	Yes	No	SR	MVB, SL	CB
10	45	Male	No	No	SR	MVB, SL	
11	58	Female	Yes	No	MVB	SR, SL	
12	57	Male	Yes	Yes	MVB	SR, SL	
13	50	Female	Yes	No	MVB	SR, SL	
14	50	Male	Yes	Yes	MVB	SR, SL	
15	53	Male	Yes	No	MVB	SR, SL	
16 (outside region)		Male					
17	54	Male	Yes	No	SL	MVB, SR	
18	40	Female	No	No	SL	MVB, SR	CB
19 (missed FG)	52	Female			CB	MVB, SR	
33 (missed FG)	31	Male	No	No	CB	MVB, SR	

FG = focus group.

### Theorisation

Theorisation was based on identification of recurrent attitudes among GPs, and GP profiles were defined by combinations of beliefs towards these attitudes. These attitudes merged from the axial coding and had been independently identified by the 4 researchers that had analysed the transcripts (CB, MVB, SR, SL). Profiles were defined by consensus among the research team. We hypothesized that the GP profiles would predict GP commitment to participation in the Diagest 3 study. Detailed results from this qualitative step will be reported in a future paper; here, we report the general findings regarding GP profiles and the distribution among the Diagest 3-GP motivation study participants.

### Step two: Cross-sectional study

A questionnaire was developed based on the results of the theoretical coding exercise, specifically, the recurring beliefs and attitudes that defined GP profiles ( Additional file 1). The questionnaire was submitted to the main study coordinator (PF) for approval, and tested on a panel of GPs not involved in the Diagest 3 study (n = 5). A grid was constructed to analyse the questionnaire answers and classify the respondent Diagest 3-GPs into the profiles defined in the theorisation step (Table 2). The questionnaire was sent to all eligible GPs of patients from the Diagest 3 study who had been lost to follow-up (n = 156, Figure 1). To further test the association between GP profile and involvement in the Diagest 3 study, a case report form (CRF) was attached to all questionnaires for completion by the GP ( Additional file 2).

**Table 2:** Analytic grid used to assign respondent GPs into defined profiles

**Profile**	**Uninterested**	**Passive**	**Slighted**	**Engaged**
Items of the questionnaire	1.1	1.3	1.3	1.3
	1.2		1.4	1.4
	2.3	2.3	2.3	2.6
	2.4	2.4	2.4	2.8
		2.5	2.6	
			2.7	
	3.1	3.1	3.4	3.4
	3.2	3.3		
	4.2	4.2	4.3	4.4
	4.3	4.3	4.5	4.71
			4.6	
			4.72	
			4.73	
	5.1	5.1	5.212	5.211
		5.223	5.213	5.221
			5.222	5.231
			5.231	
			5.232	
			5.233	
Total				
Allocated profile				

As we feared a bias due to population differences between the GPs of patients who completed the study and the GPs of patients who did not finish the study, questionn ent, social labelling, the “foot-in-the-door” technique (see below), and mailing and response monitoring.

### Layout and presentation of the questionnaire

Several described layout techniques were implemented [9,10], and enhanced to stimulate GP group-compliance, identification and internalisation [11-13]. The questionnaire was printed on both sides of an A4 size page, written in a familiar and large font (14 pt Times New Roman). Non-aggressive colours were used (black, blue and green). Only the identification notice (“Department of General Medicine”, “4 junior researchers”) and financial terms (“compensation”) were written in thick red letters. After testing with the 5 GPs from the test panel, the layout of the questionnaire was rearranged into 5 blocks to clarify its structure.

### Financial inducement and social labelling

Financial compensation is known to improve response rates [10]. This part of the study was funded by the Regional Union of Self-Employed Practitioners (URMEL) Nord Pas de Calais, which compensated GPs €.23 for completing and returning the questionnaire and CRF. This amount was chosen because of its value as a positive social label and internalisation symbol: at the time, this amount was the standard consultation fee for French medical specialists, and GP unions were negotiating for its extension to GPs, whose income is solely based on fee-for-service payments. Positive social labelling in this study involved offering GPs the same fee as a medical specialist; this positive social label carries a symbolic value of social acknowledgement. Combining labelling with a foot-in-the-door technique has been proven to increase compliance [14].

### Foot-in-the-door technique

Our hypothesis was that the act of filling out a questionnaire on their opinion of the Diagest 3 study would be a pleasant task that required a low time investment, and that it would be sufficient for GPs to engage with in the main study. For this reason, we used the questionnaire as a “foot-in-the-door” [15] to stimulate the completion of the attached CRF, which required a much higher time investment of the GPs. A foot in the door is defined as a task needing a low investment, but sufficient for the subject to make a commitment. Once committed, the consequence of the freezing effect (as defined by Kurt Lewin in 1943) is an escalation of commitment that pushes the subject to complete a much heavier task linked to the initial one that justifies the completion of the first task. The detailed analysis of the questionnaire answers will be reported in a separate publication; here, we describe the rate of questionnaire and CRF return as a measure of GP engagement in the hospital-led Diagest 3 study.

### Mailing and response monitoring techniques

Two prepaid return envelopes were attached to the mailings: one for the questionnaire and one for the CRF. Questionnaires were mailed in blocks of 7 to 30. As soon as they were sent, GPs were informed by telephone (GM, MVB, SR, QB) that they would receive a paid questionnaire that could be quickly completed. This is called the “law-ball*”* technique [2], and is used for questionnaires that may not appear long, but that the completion of which might be time consuming as the answers are not obvious. Telephone calls started with a junior researcher introducing himself as a young colleague (identification inducing sympathy [11,12]) followed up by a common greeting (the “foot-in-the-mouth” technique described by Howard [16] as a way to increase responses). Returns were monitored, and after 8 weeks, non-respondents were called again by the same researcher who proposed to send the questionnaire again. This was repeated once again to reach a maximum 3-wave mailing as described by Barclay *et al.*[17] to maximise responses.

### Step three: Process analysis

Process analysis is an action research assessment method. Its goal is not only to analyse and improve dysfunctional interactions, but also to discover new methods of collaboration between structured groups [18]. Our hypothesis was that participation in the Diagest 3-GP motivation study would modify the level of participation in the main Diagest 3 study [2] and contribute to the collection of additional patient data of comparable quality to that collected in the hospital. This analysis was based on the information collected in the CRFs ( Additional file 2), which had been attached to the questionnaires. The CRF collected 4 types of data: social data about patients lost to follow-up, biometric data from the GPs’ records, biological outcomes from blood testing and the reasons patients gave to their GP for dropping out of the main Diagest 3 study.

The main outcomes were: CRF response rates, typology of transmitted data and data quality.

### Ethical approval

Ethical approval was given for the Diagest 3-GP motivation study by the CCPPRB of Lille on Dec. 7, 2004, reference number 04/50.

## Results

### Qualitative exploratory study

Nineteen GPs were contacted and a total of 13 face-to-face interviews were conducted: 12 interviews were needed to reach data saturation and 1 additional interview was conducted to verify saturation (Table 1). Each interview lasted from 20 to 45 minutes. One GP at first refused to be interviewed, then accepted but felt upset, declaring it a waste of time. Another GP accepted the interview, but then refused because the topic was not initially made clear. Two GPs did not match the inclusion criteria (one worked outside Northern France and the other was retired). Interviews were usually conducted at the GP’s office or home.

Focus groups were attempted but were less successful: for the first focus group, we invited 6 to 8 GPs from the Lille area to meet at the Department of General Practice; however, 6 GPs declined on short notice and the group discussion took place with only 2 GPs. These interviews were therefore not considered as a focus group, and were transcribed as 2 separate interviews, analysed and pooled with the data from the face-to-face interviews to confirm data saturation (Table 1). Additional focus groups were cancelled.

Coding, triangulation and theorisation succeeded with no major problems. We switched from manual coding to software-assisted coding starting with the 10th interview. The results of 3 hand-coded transcripts that were re-analysed with the software showed no axial coding differences. In general, we reached 3 conclusions based on our analysis of the interview transcripts. First, GPs are motivated if they are involved from the outset with the hospital team in the shared management of their enrolled patients. Second, although patient education as a tool for primary prevention lies within the domain of primary care (and not the hospital), many GPs were doubtful about the usefulness, efficacy and acceptability of the education programmes in the Diagest 3 study. Finally, all interviewed GPs believed that the hospital teams consider GPs with disdain because they are not supposed to be qualified to manage pregnant women with GD. Based on these findings, we developed 4 GP profiles.

#### Uninterested

GPs showed no interest in the follow up of pregnancies and the management of GD. They quickly referred their pregnant patients to the maternal-foetal medicine (MFM) department at the hospital and did not remember their patients’ specific case. They were not concerned by the Diagest 3 study and did not motivate their patients to participate.

#### Passive

GPs felt excluded from the patient-physician relationship once they had referred their pregnant patients to the MFM department at the hospital. They did not remember what happened after referral and did not completely recall the episode of GD. They were somewhat interested in the Diagest 3 study.

#### Slighted

GPs referred their pregnant patients to the hospital MFM department because they had to, and they didn’t tolerate exclusion from the patient-physician relationship after referral. They complained about having been badly informed about the management of their patients and asked for training to share in the management of GD. Some refused to motivate their patients to follow the structured education programme in Diagest 3, feeling that they could provide patient education themselves, or they motivated their patient to advocate to the hospital team for the role of the GP in their care.

#### Engaged

Like slighted GPs, they referred their pregnant patients to the MFM department because they had to, but they asked their patients to keep in touch and they actively shared the management of their GD patients with the hospital team. Their interest in the Diagest 3 study appeared to be based on its natural continuation of patient management. As part of the research team, these GPs motivated their patients to participate in the Diagest 3 education programme.

### Cross-sectional study

**Figure 2:**
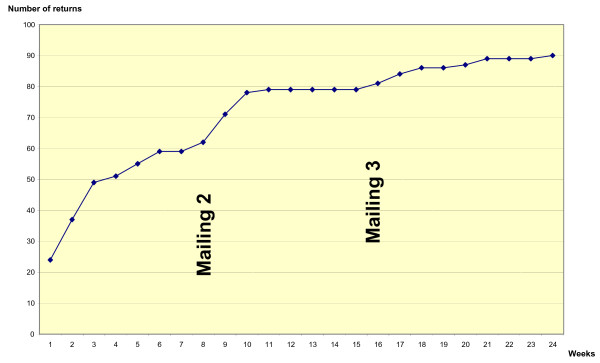
Cumulative return of questionnaires sent with attached CRF (n = 157).

**Figure 3:**
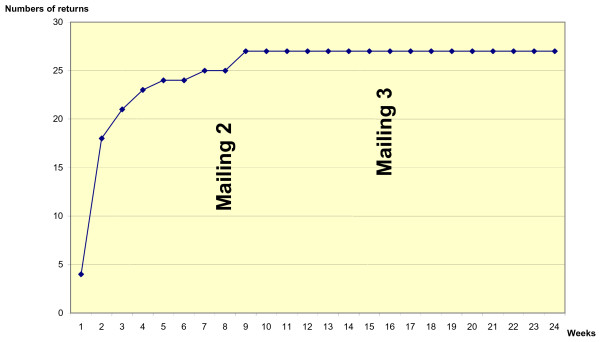
Cumulative return of questionnaires sent without a CRF (n = 37).

The highest response rate from GPs from the main Diagest 3 study at the time just prior to the GP motivation study was 32% (n = 7/22; Figure 1). In this Diagest 3-GP motivation study, the response rate to the questionnaire over 24 weeks was 57% (n = 90/157) when it was coupled to a CRF (Figure 2) and 73% (n = 27/37) when the questionnaire was sent without a CRF (control group) (Figure 3). The response time appeared to be shorter for questionnaires sent without a CRF, and the 3rd mailing was unproductive (Figure 3). No significant difference in questionnaire answers was found between the study population (n = 90) and the controls (n = 27), and questionnaires were pooled to describe GP profiles. From the 117 (90 + 27) questionnaires returned (Figure 1), 1 was lost and 5 were unable to be used for analysis (nonsense answers or insufficient completion). Thus, a total of 111 questionnaires were analysed using the profile grid developed from our qualitative exploratory study (Table 2).

**Figure 4:**
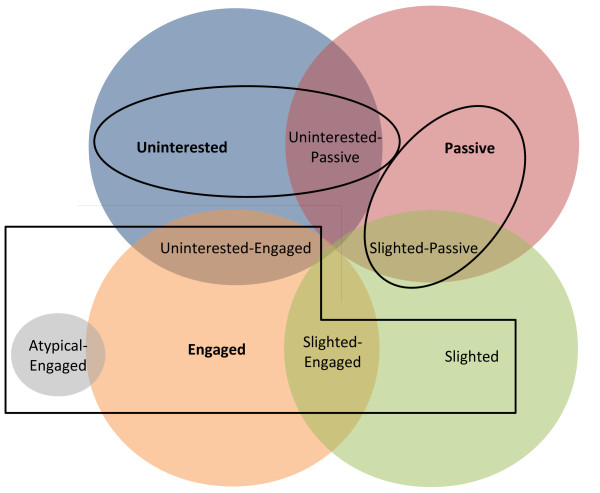
**Venn diagram illustrating the connections between the different GP profiles.** The red line delimits the engaged profile of GPs who are more likely to participate in hospital-based research (lower left side), from the passive and uninterested profiles of GPs who are less likely to participate (upper right side.

We found that with the grounded approach to profile theory, not all questionnaire answers fit within the grid (Table 2), and GPs’ answers often placed them between 2 profiles. For this reason, we expanded the analysis to include 5 overlapping profiles. The Venn diagram in Figure 4 gives a global idea of the connections between the different populations.

#### Uninterested-engaged

GPs actively delegated as many tasks as possible to the specialist and implemented all instructions from the specialist.

#### Atypical-engaged

GPs declared that they would follow up with their GD patients and implement education, but they did not remember the patient with GD or their enrolment in the Diagest 3 study.

#### Uninterested-passive

GPs described themselves as feeling excluded, but they behaved like uninterested GPs because of a reported lack of time.

#### Slighted-passive

GPs were aware of their patients’ GD and were upset about being excluded from their care, but they did not remember the Diagest 3 study.

#### Slighted-engaged

GPs remembered the study but vacillated between encouraging their patients to attend the workshops and keeping them away from the workshops because they excluded the GP from the doctor-patient relationship.

**Figure 5:**
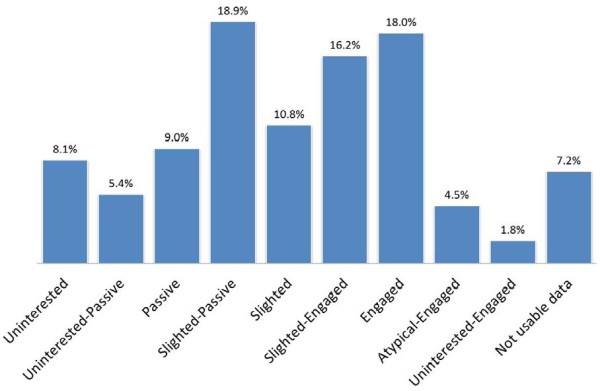
Distribution (in percentages) of GPs into the 9 GP profiles as determined by the qualitative exploratory and cross-sectional study results.

Distribution of the GPs in the 9 profiles is shown in Figure 5: uninterested (8.1%), uninterested-passive (5.4%), passive (9%), slighted-passive (18.9%), slighted (10.8%), slighted-engaged (16.2%), engaged (18%), atypical-engaged (4.5%) and uninterested-engaged (1.8%). We found that the slighted profile was not a relevant predictor for involvement in a hospital-led study. Slighted-passive GPs believed that patient education was their responsibility, but declared that they did not have the time to implement it or that it was a waste of time because their patients would not change their behaviour. These GPs also did not complete the CRF. Slighted-engaged GPs were willing to share in patient education, complementing the standard education provided in hospitals with a face-to-face approach in their practice, and they were willing to attend a training programme on the follow-up of GD patients. These GPs filled in the CRF. Uninterested-engaged GPs showed such a high degree of deference to hospital practitioners that they would comply with whatever they were asked to do by the specialists. Based on our findings, we grouped the GPs into 3 profiles (uninterested, passive and engaged), which allowed a good level of prediction of participation in the hospital-led study (red line in Figure 4). The engaged profile pooled GPs with engaged, slighted-engaged, slighted, atypical-engaged and uninterested-engaged profiles, totalling 51.3% of the GPs studied. These GPs were more likely to engage in hospital-led research and encourage their patients to follow-up with the study, whereas GPs in the uninterested group (pooled GPs with uninterested and uninterested-passive profiles) and passive group (pooled GPs with passive and slighted-passive profiles) were not. Future studies will validate these findings.

### Process analysis

A total of 90 GPs out of the 157 who were sent a questionnaire with an attached CRF returned the questionnaire, and of these, 82 also returned the attached CRF (Figure 1). One CRF was lost after reception, resulting in an answer rate of 52% (81/157). Characteristics of GPs who did not return either the questionnaire or the CRF could not be determined, as there was no clear link between GPs and their assigned patients. Six GPs did not know the patient and 9 had lost track of their patient. Thirteen patients (16%) had changed GPs, and details about their new GP were available for 3 of them. We contacted the new GPs, which allowed collection of data for 1 additional patient. A total of 28 GPs (39%) were not the GP that was identified by the patient at the time of Diagest 3 study enrolment.

On the CRF forms, responding GPs reported that 6 patients willingly withdrew from the study, 1 patient died, 7 (9%) had a new pregnancy and 3 had become diabetic. This led to useful data for 17 patients (21%), who could be excluded from the main Diagest 3 study per study protocol.

A subgroup of 35 GPs out of the 81 (43%) who returned their questionnaire and completed the attached CRF reported that they knew the patient and were still in charge of her follow-up. Of these, 33 CRFs provided usable data, representing 23% of the data in the main Diagest 3 study. Twenty-four CRFs (73%) were fully completed, representing new data for 16% (24/152) of the patients who completed the entire Diagest 3 study. Two additional completed datasets were collected directly by the main study investigators from among the subjects’ GPs.

## Discussion

It is feasible to engage field GP participation in hospital-led studies that involve their patients. Based on our interviews, when GP participation is not planned from the beginning of the study, the GPs felt excluded and disdained. Psychology of commitment motivational techniques developed by social psychologists may be of use in regaining their interest. Our survey-based approach used many of these techniques and led to response rates greater than 50%, even when a time-intensive task (i.e., filling in a CRF) is required.

As mentioned in the introduction, there have been few studies on this topic reported in the literature. Some studies have assessed the recruitment of GPs into research networks [9,18,19] or studied the attitudes of GPs towards research [3,20-22]. None have explored the commitment of field GPs to a study initiated by hospital practitioners. Nevertheless, our study confirmed that there is still a lack of interest in research and research culture [3,20,22] among some French GPs. French GPs who conduct academic work are mainly involved in pedagogy and learning; contrary to the literature [17,19], 2 GP instructors in our study did not show any greater interest in research than other GPs. We suspect that the “lack of time” stated by many GPs is actually a lack of motivation to engage in hospital-led research, as studies have shown there is no explicit link between clinical workload and trial involvement [21]. The exclusive fee-for-service remuneration of French GPs appeared to be a major barrier for participation (as shown in an Australian study comparing participation rates under a fee-for-service or capitation payment model [22]), and the stipend from the Regional Union of Self-Employed Practitioners (URMEL) was a determining incentive in getting GPs to participate [3,10]. The response rate was within the expected range [17], at the lower limit when a costly task was required and the higher one when it was not, showing that the consequences of the initial feeling of discredit were blurred by the implementation of psychosocial techniques.

We acknowledge several limitations to this study. In our qualitative exploratory study, data saturation was reached for the study population, but as the main study had initially started in the large urban district of Lille, all the interviewed GPs were working there, with loose associations between GPs and academic specialists from the 3 academic hospitals. Outcomes might have been different if interviews had involved GPs from smaller urban districts (e.g., Calais, Dunkirk, Valenciennes) where relationships between GPs and hospital specialists might be stronger. Purposive sampling might have been a more adequate approach. The fact that the interviewers and coders were GPs themselves might have presented a preconception bias, though we avoided giving them background information on the main Diagest 3 study and we ensured that the transcripts were not coded by the interviewer.

In our cross-sectional analysis, the sample size could not be computed on the main outcome (participation rate in GPs), since it depended on the main study sample size and many “regular doctors” appeared to not know the patient enrolled in the Diagest 3 study. The inclusion of GPs in our study was based on their identification by the study subjects as their “regular doctor.” This method of recruitment revealed a lack of reliability among GPs, as one-third of the GPs were not able to provide data about the patients who had identified them as their GP. As of 1 January 2006, French people must be registered on their GP’s patient list, although they may change their GP as often as they want, and some French women receive primary care from their gynaecologist rather than from their GP. Social Security is not authorized to disclose the name of the actual regular doctor of a patient (national data processing authority), and for this reason, there is still no other means by which we can track or verify a particular subject’s “regular doctor,” except to rely on identification by the subject.

The questionnaire and its analysis grid were based on the outcomes of the qualitative exploratory study. Yet, participants do not answer a questionnaire the same way they participate in a face-to-face interview: they appear to be more conformist with regard to their group attachments [11]. The first question in the questionnaire was supposed to allocate GPs to one of the 4 profiles, and the allocation then validated by their answers to subsequent questions. However, the answers given by the GPs did not follow this hypothesis, and the initial 4 profiles had to be redesigned into 9, and then redistributed on a pragmatic basis into 3, with GPs in the new engaged profile being more likely to participate in a hospital-led study. This redesign of the GP profiles prevented us from associating CRF response rates to each profile as initially planned, though a trend towards higher participation rates among the engaged GPs was observed. Our sample size was too small to conduct a factor analysis, and we had already pooled the 90 questionnaires sent with an attached CRF and the 27 sent without a CRF for the profile analysis. Future studies with larger sample sizes will include a factor analysis to support the validity of our profile analysis results.

Another weakness of this study is that the CRF data collected were retrospective, whereas those collected in the main study were prospective. This led to a higher level of missing data in our study. Nevertheless, the average value of each item collected on the CRF and its confidence interval were consistent with the values collected in the main study, indicating that the data collected on the CRF in our motivation study were of good quality. For example, the quality of data for blood pressure collected on the CRFs was assessed against data from the ESCAPE study [23]; measurements fell within the same range as that of other blood pressure studies in general practice in France.

The findings of the Diagest 3-GP motivation study will be useful as we design future hospital-led studies in which engagement of field GPs is needed. Specifically, we propose that at the enrolment of each subject, GPs are included in follow-up, and receive special training and financial compensation. Depending on the GP’s level of engagement, we also propose that a questionnaire similar to the one used for this study be developed based on the integrative model of behavioural prediction developed by Martin Fishbein [24]. The questionnaire would be submitted to each GP and the data used to validate our typology. If our findings are validated in future studies, then we would recommend that methods to engage field GPs be included in the protocols of studies where the field GP is needed to lower the subjects’ drop-off rate.

## Conclusions

The most compelling finding of this study is that field GPs that fit the engaged profile are more likely to participate in a hospital-led study than GPs that fit either the uninterested or passive profiles. Further studies are necessary to verify this finding, and our team is working to combine this model with the integrative model of behavioural prediction developed by Martin Fishbein [24]. With regard to engagement of GPs in hospital-led research in which their patients are enrolled, it is important that the participation of GPs is planned at the outset. Including GPs in the study design as early as the time of patient enrolment can avoid feelings of exclusion, hostility and resignation among GPs. Further studies will determine the predictive value of GP profiles for engagement in hospital-led research. A second important finding of our work is that psychology of commitment motivational techniques—including layout and presentation of the CRF, financial inducement, response monitoring, social labelling, identification and symbolic internalisation—may be useful in regaining the interest of GPs in the hospital-led study. Under these conditions, we demonstrated that 1 out of 2 GPs were compelled to participate by returning their questionnaire and completed CRF, leading to a significant increase in patient data. To achieve identification and internalisation among GPs, it might be useful for an academic GP, who shares the attitudes of primary care practitioners and the engagement in research of hospital-based colleagues, to act as an interface between the hospital research steering committee and the GPs of enrolled patients.

## Abbreviations

CFR, Case report form; GP, General practitioner; MFM, Maternal-foetal medicine; T2D, Type 2 diabetes; URMEL, Regional Union of Self-Employed Practitioners.

## Competing interests

All the authors declare no competing interests in the field of this work.

## Authors’ contributions

CB directed and supervised the Diagest-GP motivation study and wrote the manuscript. MVB, SR and SL conducted the interviews, coded the transcripts, and analysed the questionnaire data with GM. SR and SL wrote the French preliminary redaction on the qualitative exploratory study. MVB, GM and QB managed the questionnaires and the clinical reports. MVB and GM wrote the French preliminary redaction on the cross-sectional study. QB wrote the French preliminary redaction on the process analysis. AV is responsible of the entire Diagest research programme. PF is the main investigator and coordinator of the Diagest 3 study. All authors read and approved the final manuscript.

## Pre-publication history

The pre-publication history for this paper can be accessed here:

http://www.biomedcentral.com/1471-2296/13/63/prepub

## Supplementary Material

Additional file 1 **Codification of items of the Diagest 3-GP questionnaire as applying toTable** 2. Click here for file

Additional file 2 **English translation of the case report form, as it was sent to the usual GP of lost to view subjects enrolled in the Diagest 3 study.** Unless this patient has retired her consent to the Diagest 3 study, this data are usual management data for persons at high risk of developing type 2 diabetes. No specific consent of the patient is required. Click here for file
