# Supplementary material for: Enhancing field GP engagement in hospital-based studies. Rationale, design, main results and participation in the diagest 3-GP motivation study
Source: BMC Fam Pract. 2012 Jun 21;13:63. doi: 10.1186/1471-2296-13-63 (PMC3441219; doi:10.1186/1471-2296-13-63)
Supplement: Additional file 1 — Codification of items of the Diagest 3-GP questionnaire as applying toTable 2. [file 1471-2296-13-63-S1.doc]

Table 1: Characteristics of interviewed GPs and the interview approach.

| **GP number** | **Age** | **Gender** | **Continuous education** | **research** | **Interviewer** | **Coding** | **3rd coding** |
| --- | --- | --- | --- | --- | --- | --- | --- |
| 1 (retired) |  | Male |  |  |  |  |  |
| 2 | 52 | Male | Yes | No | SL | MVB, SR |  |
| 3 (refused interview) |  | Male |  |  |  |  |  |
| 4 (gynaecologist) |  | Female |  |  |  |  |  |
| 5 | 56 | Male | No | No | SL | MVB, SR |  |
| 6 (refused to answer) |  | Male |  |  | SR |  |  |
| 7 | 47 | Male |  |  | SR | MVB, SL |  |
| 8 | 46 | Male | No | No | SR | MVB, SL |  |
| 9 | 39 | Female | Yes | No | SR | MVB, SL | CB |
| 10 | 45 | Male | No | No | SR | MVB, SL |  |
| 11 | 58 | Female | Yes | No | MVB | SR, SL |  |
| 12 | 57 | Male | Yes | Yes | MVB | SR, SL |  |
| 13 | 50 | Female | Yes | No | MVB | SR, SL |  |
| 14 | 50 | Male | Yes | Yes | MVB | SR, SL |  |
| 15 | 53 | Male | Yes | No | MVB | SR, SL |  |
| 16 (outside region) |  | Male |  |  |  |  |  |
| 17 | 54 | Male | Yes | No | SL | MVB, SR |  |
| 18 | 40 | Female | No | No | SL | MVB, SR | CB |
| 19 (missed FG) | 52 | Female |  |  | CB | MVB, SR |  |
| 33 (missed FG) | 31 | Male | No | No | CB | MVB, SR |  |

FG=focus group.
