# Supplementary material for: Enhancing field GP engagement in hospital-based studies. Rationale, design, main results and participation in the diagest 3-GP motivation study
Source: BMC Fam Pract. 2012 Jun 21;13:63. doi: 10.1186/1471-2296-13-63 (PMC3441219; doi:10.1186/1471-2296-13-63)
Supplement: Additional file 2 — English translation of the case report form, as it was sent to the usual GP of lost to view subjects enrolled in the Diagest 3 study. Unless this patient has retired her consent to the Diagest 3 study, this data are usual management data for persons at high risk of developing type 2 diabetes. No specific consent of the patient is required. [file 1471-2296-13-63-S2.doc]

Table 2: Analytic grid used to assign respondent GPs into defined profiles.

| Profile | Uninterested | Passive | Slighted | Engaged |
| --- | --- | --- | --- | --- |
| Items of the questionnaire | 1.1  1.2  2.3  2.4  3.1  3.2  4.2  4.3  5.1 | 1.3  2.3  2.4  2.5  3.1  3.3  4.2  4.3  5.1  5.223 | 1.3  1.4  2.3  2.4  2.6  2.7  3.4  4.3  4.5  4.6  4.72  4.73  5.212  5.213  5.222  5.231  5.232  5.233 | 1.3  1.4  2.6  2.8  3.4  4.4  4.71  5.211  5.221  5.231 |
| Total |  |  |  |  |
| Allocated profile |  |  |  |  |
